# Neural Dynamics of Attentional Boost Effect

**DOI:** 10.1002/brb3.71250

**Published:** 2026-02-10

**Authors:** Xintong Chen, Xuan Lyu, Li Zhu, Qin Cui, Qing Yang, Xinglin Li, Kaiye Xiang, Chun Zheng, Chao Fu

**Affiliations:** ^1^ School of Education Qinghai Normal University Xining Qinghai P.R. China; ^2^ School of Nursing Qingdao University Qingdao P.R. China; ^3^ School of Education Qinghai Minzu University Xining Qinghai P.R. China; ^4^ Qinghai Plateau Brain Science Research Center Xining Qinghai P.R. China; ^5^ Qinghai Cardio‐Cerebrovascular Specialty Hospital Qinghai High Altitude Medical Research Institute Xining Qinghai P.R. China

**Keywords:** attentional boost effect, distractor inhibition, event‐related potentials, old/new effect, target enhancement

## Abstract

**Background**: The attentional boost effect refers to enhanced memory for information presented concurrently with target detection. This study explored the temporal neural dynamics of attentional boost effect during encoding and recognition to clarify whether it results from target enhancement or distractor inhibition.

**Methods**: A dual‐task paradigm combining digit detection with word memorization was employed. Electroencephalography was recorded throughout the encoding and recognition stages. Event‐related potential components, including P1, N1, P2, N2, P3, FN400, and late positive component (LPC), were analyzed across four regions of interest to track the time course of neural processing associated with attentional facilitation.

**Results**: Recognition accuracy for target‐associated words was significantly higher than for baseline and distractor words, confirming a robust attentional boost effect. Event‐related potential analyses revealed that during the encoding stage, target words elicited shorter P2/N2 latencies and larger P3 amplitudes. During the recognition stage, target words evoked more positive FN400 and LPC responses relative to new words.

**Conclusions**: The attentional boost effect emerges from the combined operation of target enhancement and distractor inhibition. These findings outline the neural timeline of attentional boost effect and provide insights for developing cognitive enhancement and rehabilitation strategies.

## Graphical abstract

An ERP study reveals a distinct neural timeline of enhanced processing for target‐paired stimuli, from encoding to recognition, supporting the mechanism of target enhancement over distractor inhibition.

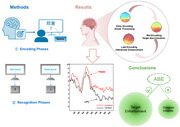

## 1 Introduction

The relationship between attention and memory remains a central topic in cognitive psychology (Backer and Alain 2013; Chun and Turk‐Browne 2007). According to the theory of limited attentional resources, attention is a finite cognitive capacity, and memory performance declines when multiple tasks compete for these limited resources (Kinchla 1992). However, this view was challenged by Swallow and Jiang (2010), who demonstrated that performing a memory task concurrently with a target detection task can enhance, rather than impair, memory performance—specifically for information presented simultaneously with a detection target. This finding suggests that under certain conditions, divided attention may facilitate memory encoding. Swallow and Jiang termed this phenomenon the attentional boost effect (ABE). Subsequent studies have extended the ABE across various domains of memory, including short‐term memory (Spataro et al. 2020), implicit memory (Chen et al. 2021; Spataro et al. 2013), and source memory (Mulligan et al. 2016, 2021). Moreover, the ABE has been observed across different types of materials, such as faces (Swallow and Jiang 2012) and words (Mulligan et al. 2014). Collectively, this body of evidence highlights the robustness and generalizability of the ABE as a stable cognitive phenomenon.

To account for the mechanisms underlying the ABE, Swallow and Jiang (2013) proposed the dual‐task interaction model. This model extends the theory of limited attentional resources by suggesting that attentional allocation is flexible rather than strictly capacity‐bound in dual‐task contexts. Specifically, when perceptual information from target and distractor stimuli accumulates to a decision threshold that allows for their discrimination, individuals execute a behavioral response (e.g., button press or internal counting). This response triggers a time‐based selective attention mechanism, typically accompanied by phasic activation of the locus coeruleus–norepinephrine (LC–NE) system, which induces a transient enhancement of neural activity (Aston‐Jones and Cohen 2005; Yebra et al. 2019). The resulting surge in norepinephrine is thought to broadly modulate cortical sensory regions, thereby facilitating the perceptual processing of stimuli presented concurrently with the target and producing the ABE. Empirical evidence supports this model: distractors with high perceptual similarity to targets fail to elicit the ABE (Swallow and Jiang 2014b), and increased perceptual or semantic load prior to target decision‐making does not diminish the effect (Swallow and Jiang 2014a; Zheng et al. 2021). These findings reinforce the dual‐task interaction framework, indicating that the ABE primarily arises from the target decision‐making process rather than from stimulus‐driven factors alone.

Recently, event‐related potential (ERP) techniques, which provide high temporal resolution, have been employed to investigate the neural mechanisms underlying the ABE. Jing (2015) first reported that target conditions elicited larger P3 amplitudes in mid‐posterior brain regions than distractor and baseline conditions, suggesting that the P3 reflects processing enhancement triggered by target detection. To further examine this effect, Lin (2019a) manipulated the target‐to‐distractor (TD) frequency ratio (high‐frequency target group: TD = 4:1; low‐frequency target group: TD = 1:4) to explore how target frequency, reflecting the novelty of detection stimuli, modulates the P3 component. Across both groups, low‐frequency stimuli consistently evoked larger P3 amplitudes than high‐frequency stimuli, leading the authors to propose that the P3 component observed in the ABE reflects the novelty of the detection stimulus. Subsequent ERP study conducted by Lin et al. (2020) examined the neural dynamics of encoding stage. Their results identified a distinct temporal pattern across three encoding stages: In the early encoding stage, target words (TW) and distractor words (DW) elicited comparable P1 and N1 amplitudes, suggesting similar early sensory processing. During the mid‐encoding stage, DW evoked a larger N2 component than TW, a difference tentatively attributed to response inhibition. In the late encoding stage, TW elicited significantly larger P300 amplitudes over central‐parietal regions compared to both DW and baseline words (BW). Given that the P300 is a well‐established neural marker of attentional resource allocation, these findings indicate that target detection triggers a generalized attentional enhancement. This leads to increased cognitive resource allocation to concurrently presented background information, thereby supporting the target enhancement hypothesis of the ABE.

Previous research has consistently demonstrated that selective attention operates through a dual mechanism involving both the activation of target information and the inhibition of distracting information. Accordingly, the ABE may arise either from enhanced processing of stimuli presented concurrently with attended targets—known as the target enhancement hypothesis—or from reduced memory for background information due to the active suppression of distractors (Meng et al. 2019; Sisk and Lee 2024), referred to as the distractor inhibition hypothesis. While most prior studies have primarily focused on the target enhancement aspect of the ABE, using the P3 component as its neural correlate (Jing 2015; Lin 2019a; Lin et al. 2020), research on the distractor inhibition mechanism—particularly regarding the N2 component—remains limited and inconsistent. For instance, whereas Lin (2019b) and Lin et al. (2020) found that distractor conditions elicit larger N2 amplitudes than target conditions, Duan (2021) reported the opposite pattern, with target words eliciting larger N2 amplitudes. To address this gap, the present study employs ERP techniques to investigate the dynamic neural processes underlying the ABE across both the encoding and recognition stages. Specifically, we examine target enhancement, indexed by the P3 component, and response inhibition, indexed by the N2 component. In addition, given prior evidence that stimulus frequency may influence the ABE (Duncan‐Johnson and Donchin 1977; Lin 2019a), we manipulated the frequency of distractor stimuli (using a 4:1 ratio of high‐frequency distractor words (HFDW) to low‐frequency distractor words (LFDW); see Section 2 for details) to explore how stimulus frequency modulates distractor inhibition. This within‐distractor comparison offers a more precise test of inhibitory processes than prior designs.

During the encoding stage, given that early encoding ERP components (P1 and N1)—known to originate from the parieto‐occipital pathway and reflect early visual selective attention—have not been shown to differ between target and distractor conditions in prior ABE research (Lin et al. 2020; Luck et al. 2000), we hypothesized that P1 and N1 amplitudes would not differ significantly between TW and DW, nor between LFDW and HFDW in our study. Regarding mid‐encoding ERP components P2 and N2, prior research indicates that the P2—linked to anterior cingulate and prefrontal activity—reflects attentional maintenance or the detection of expectancy violations, as seen in larger amplitudes for No‐Go stimuli in inhibition tasks (Johnstone et al. 2005). The N2, generated in similar regions, is associated with conflict monitoring and response inhibition, typically showing more negative amplitudes under conflict or error conditions (Chao Fu et al. 2018; Donkers and van Boxtel 2004; Larson et al. 2014). In the present study, participants were required to respond to target stimuli while inhibiting responses to distractors. Based on the distractor inhibition hypothesis, which attributes reduced recognition of distractor‐paired words to inhibitory processing during encoding, we hypothesized that DW would elicit larger P2 and N2 amplitudes than TW. In addition, given evidence that stimulus frequency modulates processing depth and attentional resource allocation (Duncan‐Johnson and Donchin 1977). If the N2 component indeed reflects active response suppression, then HFDW—which elicit stronger automatic processing—should require greater inhibitory control. We therefore hypothesized that HFDW would elicit larger P2 and N2 amplitudes than LFDW. As for the late encoding ERP component P3, previous studies have linked it to the frontoparietal attention network and the temporoparietal junction, reflecting attentional resource allocation and memory updating (Kok 1990, 2001; Shitova et al. 2017). Low‐frequency stimuli are known to capture greater attentional resources and typically evoke larger P3b amplitudes (Donchin and Coles 1988; Polich 2007). Consistent with prior ABE‐related ERP studies reporting greater P3 amplitudes for targets than distractors (Duan 2021; Jing 2015; Lin 2019a), we hypothesized that TW would elicit larger P3 amplitudes than both DW and BW, and that LFDW would elicit larger P3 amplitudes than HFDW.

During the recognition stage, at the behavioral level, given that the ABE is characterized by enhanced memory performance for information presented concurrently with target stimuli—relative to that presented with distractor or baseline stimuli (Swallow and Jiang 2010)—we hypothesized that recognition accuracy would be higher for TW than for BW and DW, and that recognition accuracy would be higher for HFDW than for LFDW. At the neural level, two ERP components are known to be closely associated with recognition memory (Donchin and Coles 1988; Polich 2007). The first is the FN400, a negative‐going potential distributed over the fronto‐central regions, typically peaking between 300 and 500 ms after stimulus onset. The FN400 is generally regarded as an index of rapid, automatic, familiarity‐based recognition—the subjective sense of “having seen something before” without the retrieval of specific contextual details such as time or location (Meng and Guo 2009). Because new words are semantically unfamiliar, the brain must engage in more effortful processing to attempt their integration into existing semantic networks. Consequently, new items typically elicit more negative FN400 amplitudes than old items (Curran 2000; Lin et al. 2020). The second component is the late positive component (LPC)—also referred to in some literature as the P600 (Lin et al. 2020)—a positive‐going waveform most prominent over centro‐parietal regions, peaking around 500–800 ms or later. The LPC is associated with conscious, effortful, and detail‐rich episodic retrieval, supporting the recollection of both the item and its contextual attributes (Rugg and Curran 2007). Generally, old items elicit larger LPC amplitudes than new items. Given that HFDW undergo more extensive processing during the encoding stage than LFDW, they are likely to elicit a greater sense of familiarity during the recognition stage. We hypothesized that old stimuli (particularly the TW) and HFDW would elicit larger LPC amplitudes, whereas new stimuli and LFDW would evoke more negative FN400 amplitudes.

## 2 Methods and Procedures

### 2.1 Participants

A power analysis conducted using G*Power 3.1 (Faul et al. 2009; Faul et al. 2007), based on effect sizes reported in previous studies (Jing 2015; Lin 2019a; Lin et al. 2020) (*η_p_* ^2^ = 0.10–0.20), indicated that a minimum of 35 participants would be required to detect a large effect in a within‐subjects repeated‐measures ANOVA (*α* = 0.05, power = 0.95). To ensure sufficient statistical power for analyzing multiple ERP components and to account for potential attrition, 60 right‐handed junior students (30 females; 13.97 ± 0.06 years old) were recruited. All participants were right‐handed according to the Edinburgh Handedness Inventory (Oldfield 1971), had normal or corrected‐to‐normal vision, and reported no history of neurological or psychiatric disorders. Written informed consent was obtained from all participants and their parents. The study protocol was approved by the Ethics Committee of Qinghai Normal University and conducted in accordance with the Declaration of Helsinki and the ethical guidelines of the American Psychological Association. Participants received a small monetary compensation for their time.

### 2.2 Materials

Prior to the experiment, we selected 1500 two‐character words from the “Table of Low‐Frequency Word Units” in the Modern Chinese Frequency Dictionary (1986). These words were then rated by 146 college students on valence, arousal, dominance, approachability, and familiarity. After excluding words that deviated by more than 3 standard deviations from the mean on any dimension, we established a final pool of 1000 low‐frequency (0.009% ± 0.04%), emotionally neutral two‐character words (valence: 2.93 ± 1.02; arousal: 2.88 ± 0.96; dominance: 2.79 ± 0.98; approachability: 2.83 ± 1.02; familiarity: 3.29 ± 1.14). The study utilized 274 words from the pool, which were randomly assigned to the four conditions (BW, TW, HFDW, LFDW). This total included 15 practice trials and 259 formal trials (comprising 175 old and 84 new words). Statistical analyses confirmed no significant differences across the words on any of the rated dimensions.

### 2.3 Programs

**FIGURE 1:**
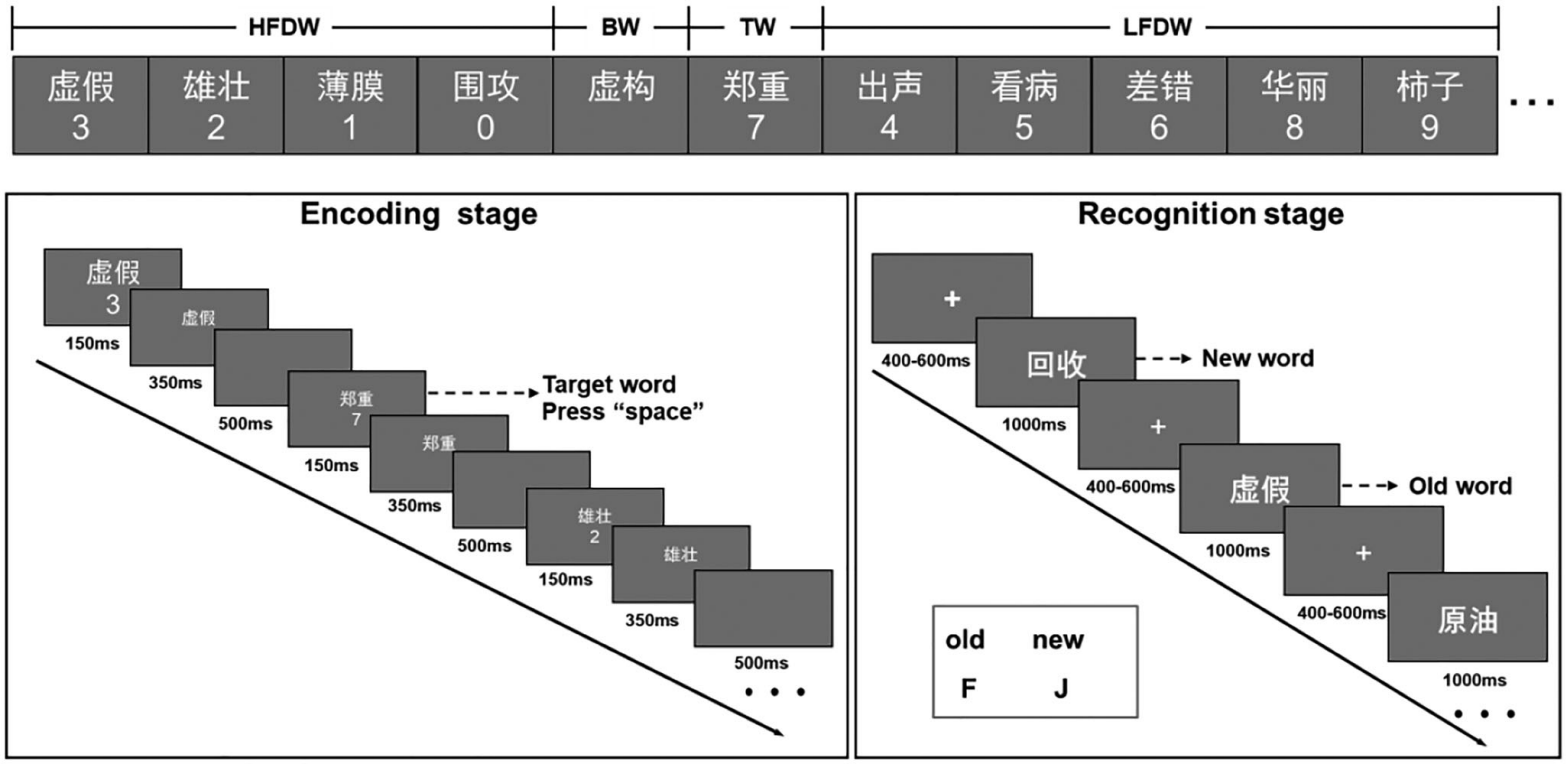
Flowchart of the experimental procedure. During the encoding stage, digits and words were presented simultaneously for 150 ms. The digits then disappeared, while the words remained on the screen for an additional 350 ms, followed by a 500 ms blank screen (inter‐stimulus interval). Participants were instructed to memorize the words while performing a concurrent target detection task—pressing the spacebar when the target digit “7” appeared and withholding responses to all other digits. During the recognition stage, old and new words were presented randomly for 1000 ms, during which participants made an old/new judgment by pressing the “F” key for old words (previously presented during encoding stage) and the “J” key for new words (not presented during encoding stage). Each trial was followed by a 400–600 ms inter‐stimulus interval. Failure to respond within the 1000 ms window was recorded as an incorrect response. BW, baseline words; HFDW, high‐frequency distractor words; LFDW, low‐frequency distractor words; NW, new words; TW, target words.

The experiment consisted of two stages: an encoding stage and a recognition stage (Figure 1). During the encoding stage, participants were instructed to memorize words presented on the screen while simultaneously performing a target detection task. Specifically, they pressed the spacebar when the target digit “7” appeared and withheld responses to all other digits. During the recognition stage, participants completed a recognition task involving the words presented during encoding. They pressed the “F” key for old words (previously presented during encoding stage) and the “J” key for new words (not presented during encoding stage).

During the encoding stage (Table 1), the words to be memorized were divided into four categories: BW (25 items), which were not paired with any digits and thus represented the focused attention condition requiring only the memory task; TW (25 items), each paired with the target digit “7”; HFDW (100 items in total), each paired with one of the high‐frequency distractor digits (3, 2, 1, or 0), with each digit appearing 25 times; and LFDW (25 items in total), each paired with one of the low‐frequency distractor digits (4, 5, 6, 8, or 9), with each digit appearing five times. In contrast to the BW, the TW, HFDW, and LFDW all involved divided attention, as participants were required to perform both the memory and detection tasks simultaneously. During the recognition stage, not all words from the encoding stage were presented to minimize experimental duration. Following Sperling's partial‐report paradigm (Sperling 1960), 84 words were randomly selected from the BW, TW, HFDW, and LFDW categories as old words, along with an equal number of new words that had not appeared during encoding. Participants were then required to perform an old/new recognition test to assess memory performance. Before the formal experiment, 15 practice trials were administered to familiarize participants with the task. During the encoding stage, digits and words were presented simultaneously for 150 ms, after which the digits disappeared while the words remained on the screen for an additional 350 ms. A 500 ms blank screen (inter‐stimulus interval) followed before the next trial began. During the recognition stage, old and new words were presented randomly for 1000 ms, during which participants were required to make an old/new judgment; failure to respond within the 1000 ms window was recorded as an incorrect response. Each trial was followed by a 400–600 ms inter‐stimulus interval. The experiment was programmed using PsychoPy3 (Peirce 2007, 2008), and stimuli were displayed on a 19‐inch color LCD monitor with a resolution of 1920 × 1080. The entire session lasted approximately 30 min.

**TABLE 1:** Word types and presentation parameters in the encoding and recognition stage.

	Word type				
	Encoding stage/Old words				Recognition stage/ND
	BW	TW	HFDW	LFDW	
Digits accompanying words	—	7	3, 2, 1, 0	4, 5, 6, 8, 9	—
Number of words (encoding stage)	25	25	4 (digits) × 25 (times)/digit = 100	5 (digits) × 5 (times)/digit = 25	—
Number of words (recognition stage)	12	12	48	12	84

Abbreviations: BW, baseline words; HFDW, high‐frequency distractor words; LFDW, low‐frequency distractor words; NW, new words; TW, target words.

### 2.4 EEG Data Acquisition and Preprocessing

EEG data were recorded using a 64‐channel electrode cap (Quik‐Cap, NeuroScan Inc.) positioned according to the extended International 10–20 system. Signals were acquired through a NeuroScan SynAmps2 amplifier and the Curry 8 acquisition software (Compumedics NeuroScan, Charlotte, NC, USA). During recording, the reference electrode was placed on the right mastoid. Vertical and horizontal electrooculograms (VEOG and HEOG) were simultaneously recorded to monitor ocular artifacts. Electrode impedance was maintained below 5 kΩ. EEG data were sampled at 1000 Hz with a bandpass filter of 0.05–100 Hz (AC coupled). Offline preprocessing was conducted using the EEGLAB toolbox (version 2024.2) and custom MATLAB (R2023b) scripts. The preprocessing pipeline included the following steps: (1) Data import: Continuous data were imported from Curry files and converted to EEGLAB format. (2) Channel location assignment: Electrode positions were assigned based on the extended 10–20 system using a custom location file. (3) Downsampling: Data were downsampled from 1000 to 500 Hz to reduce computational load. (4) Filtering: A high‐pass filter at 0.1 Hz and a low‐pass filter at 45 Hz were applied to remove slow drifts and high‐frequency noise, respectively. A 50 Hz notch filter (48–52 Hz) was applied to suppress line noise. All filters were implemented using a zero‐phase shift design to prevent temporal distortion, with a roll‐off rate of 24 dB/octave to ensure sharp frequency cutoffs and effective noise attenuation. (5) Re‐referencing: Data were re‐referenced from the right mastoid to the average of M1 and M2. (6) Non‐EEG channel removal: Channels unrelated to EEG analysis (e.g., EOG, EKG, EMG) were removed. (7) Bad channel and segment rejection (manual): Continuous data were visually inspected, and bad channels were interpolated using spherical splines. Segments contaminated by artifacts were excluded. (8) Independent component analysis (ICA): ICA was performed using the extended INFOMAX algorithm. Ocular and muscular artifacts were identified and removed using the ADJUST toolbox, with a threshold of 0.9 for “Eye” and “Muscle” components. (9) Epoching and baseline correction: Data were segmented into epochs from –200 to 1000 ms relative to stimulus onset. Baseline correction was applied using the –200 to 0 ms interval. (10) Automatic epoch rejection: Epochs exceeding ±75 µV were automatically rejected. (11) ERP averaging: Artifact‐free epochs were averaged separately for each condition to obtain ERPs.

### 2.5 Experimental Design and Statistical Analysis

Based on previous research (Lin et al. 2020) and the relationship between scalp distribution and electrode positioning, four regions of interest (ROIs) comprising 12 representative electrodes were selected for EEG analysis: Frontal (ROI1: F3, Fz, F4), Central (ROI2: C3, Cz, C4), Parietal (ROI3: P3, Pz, P4), Occipital (ROI4: O1, Oz, O2). Drawing on prior findings (Lin et al. 2020; Twomey et al. 2015; Yun et al. 2011) and waveform inspection, the encoding‐stage analysis focused on the mean amplitude and latency of five ERP components: P1 (mean amplitude: 110–170 ms; latency: 100–170 ms), N1 (mean amplitude: 130–220 ms; latency: 150–200 ms), P2 (mean amplitude: 200–300 ms; latency: 200–300 ms), N2 (mean amplitude: 250–350 ms; latency: 250–350 ms), P3 (mean amplitude: 350–650 ms; latency: 350–650 ms). For the recognition stage, analyses focused on the mean amplitudes of two ERP components: FN400 (300–500 ms) and LPC (500–1000 ms). The experimental design for the encoding stage was a 4 (word type: BW, TW, HFDW, LFDW) × 4 (ROI: frontal, central, parietal, occipital) within‐subjects two‐factor design. For the recognition stage, a 2 (recognition outcome: correct, incorrect) × 5 (word type: BW, TW, HFDW, LFDW, NW) × 4 (ROI: frontal, central, parietal, occipital) within‐subjects three‐factor design was used. Statistical analyses were performed using repeated‐measures ANOVA. All descriptive data are presented as arithmetic means ± standard errors. Statistical significance was set at *p* < 0.05. Greenhouse–Geisser corrections were applied for sphericity violations, and all pairwise comparisons were Bonferroni‐adjusted. Partial eta‐squared (*η_p_* ^2^) values were reported as measures of effect size.

## 3 Results

### 3.1 Behavioral Results

**FIGURE 2:**
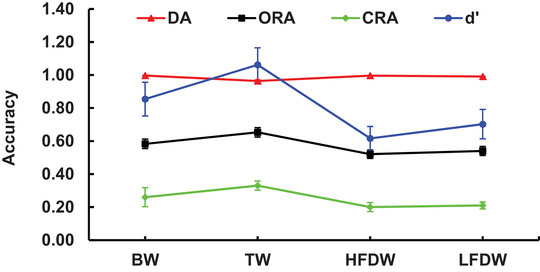
Detection accuracy during the encoding stage and recognition accuracy during the recognition stage. CRA was calculated as the hit rate for old words minus the false alarm rate for new words, and *d′* was computed as the *z*‐score of the hit rate minus the *z*‐score of the false alarm rate. Error bars represent the SEM. BW, baseline words; CRA, corrected recognition accuracy; *d*′, discriminability index based on signal detection theory; DA, detection accuracy during the encoding stage; HFDW, high‐frequency distractor words; LFDW, low‐frequency distractor words; ORA, original recognition accuracy; SEM, standard error of the mean; TW, target words.

The detection accuracy during the encoding stage and the recognition accuracy for each word type in the recognition stage are shown in Figure 2. For statistical analysis, the recognition accuracy for HFDW was calculated as the mean accuracy of words paired with high‐frequency distractor digits (0, 1, 2, 3), whereas the recognition accuracy for LFDW was derived from words paired with low‐frequency distractor digits (4, 5, 6, 8, 9). Subsequent analyses focused on the recognition accuracy of the four word types: BW, TW, HFDW, and LFDW.

#### 3.1.1 Detection Accuracy During the Encoding Stage

A one‐way repeated‐measures ANOVA with four levels (word type: BW, TW, HFDW, LFDW) on detection accuracy revealed a significant main effect of word type, *F* (1.332, 78.588) = 17.00, *p < *0.001, *η_p_^2^* = 0.22. Pairwise comparisons showed that detection accuracy for TW (0.96 ± 0.01) was significantly lower than for BW (1.00 ± 0.00, *p* < 0.001), HFDW (1.00 ± 0.00, *p* < 0.001), and LFDW (0.99 ± 0.00, *p* < 0.001), detection accuracy for BW was significantly higher than for LFDW (*p* = 0.04), but not significantly different from HFDW (*p* = 0.72).

#### 3.1.2 Original Recognition Accuracy (ORA) in the Recognition Stage

A one‐way repeated‐measures ANOVA with four levels (word type: BW, TW, HFDW, LFDW) on ORA revealed a significant main effect, *F* (3, 177) = 14.53, *p* < 0.001, *η_p_* ^2^ = 0.20. Pairwise comparisons showed that ORA for TW (0.65 ± 0.03) was significantly higher than for HFDW (0.52 ± 0.03, *p* < 0.001) and LFDW (0.54 ± 0.03, *p* < 0.001), and marginally higher than for BW (0.58 ± 0.03, *p* = 0.068). ORA for BW was also significantly higher than for HFDW (*p* = 0.003) and LFDW (*p* = 0.03). No significant difference was observed in ORA values between HFDW and LFDW (*p* = 0.46).

#### 3.1.3 Corrected Recognition Accuracy (CRA) and Discriminability Index (*d*′) in the Recognition Stage

To account for potential effects of false alarms, the CRA and the discriminability index (*d′*) were calculated following prior studies (Pan et al. 2024). Specifically, CRA was calculated as the hit rate for old words minus the false alarm rate for new words, and *d′* was computed as the *z*‐score of the hit rate minus the *z*‐score of the false alarm rate. The same repeated‐measures ANOVA as above was conducted on both CRA and *d′* values. CRA: A one‐way repeated‐measures ANOVA with four levels (word type: BW, TW, HFDW, LFDW) revealed a significant main effect of word type, *F*(3, 177) = 14.86, *p* < 0.001, *η_p_* ^2^ = 0.20. Pairwise comparisons showed that CRA for TW (0.33 ± 0.03) was significantly higher than for BW (0.26 ± 0.03, *p* = 0.01), HFDW (0.20 ± 0.02, *p < *0.001), and LFDW (0.21 ± 0.02, *p* < 0.001). CRA for BW was also significantly higher than for HFDW (*p* = 0.002) and LFDW (*p* = 0.03). No significant difference was observed in CRA values between HFDW and LFDW (*p* = 0.43). **Discriminability index (*d′*)**: A one‐way repeated‐measures ANOVA with four levels (word type: BW, TW, HFDW, LFDW) revealed a marginally significant main effect of word type, *F* (3, 177) = 13.65, *p < *0.001, *η_p_* ^2^ = 0.19. Pairwise comparisons showed that *d'* for TW (1.06 ± 0.10) was significantly higher than for BW (0.85 ± 0.10, *p* = 0.01), HFDW (0.62 ± 0.07, *p* < 0.001), and LFDW (0.70 ± 0.09, *p* < 0.001). Furthermore, *d'* for BW was significantly higher than for both HFDW (*p* < 0.001) and LFDW (*p* = 0.04). No significant difference was found in *d'* values between HFDW and LFDW (*p* = 0.20).

### 3.2 EEG Results in Encoding Stage

#### 3.2.1 ERP Components in Early Encoding Stage

**FIGURE 3:**
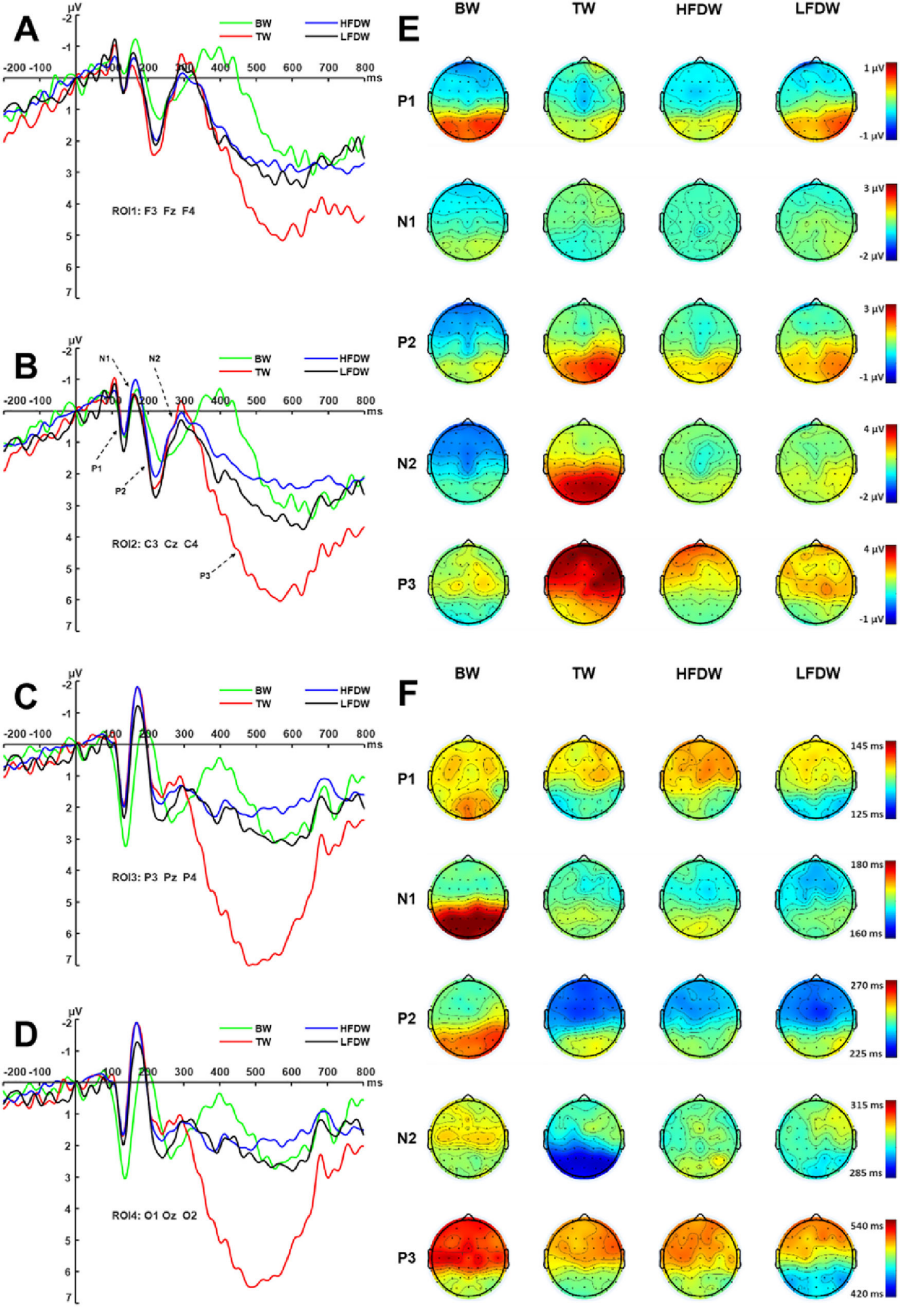
Grand‐averaged ERP waveforms and scalp topographies during the encoding stage. Panels A–D show the grand‐averaged ERP waveforms for the four word types (BW, TW, HFDW, and LFDW) across four regions of interest (ROI1–ROI4). The x‐axis represents time (ms), and the y‐axis represents ERP amplitude (µV). Panels E and F depict the scalp topographies of mean amplitudes and peak latencies, respectively, for five ERP components. Warm colors indicate positive potentials, whereas cool colors indicate negative potentials. BW, baseline words; HFDW, high‐frequency distractor words; LFDW, low‐frequency distractor words; TW, target words.

**P1 Amplitude**: A two‐way repeated‐measures ANOVA with factors word type (BW, TW, HFDW, LFDW) and ROI (frontal, central, parietal, occipital) was conducted on the mean amplitude of the P1 component (110–170 ms). The analysis revealed a significant main effect of word type (Figure 3), *F*(3, 177) = 3.65, *p* = 0.01, *η_p_* ^2^ = 0.06. Pairwise comparisons showed that the P1 amplitude for BW (0.95 ± 0.37 µV) was significantly higher than that for HFDW (−0.03 ± 0.26 µV, *p* = 0.01) and LFDW (0.13 ± 0.31 µV, *p* = 0.02), and was marginally higher than that for TW (0.09 ± 0.31 µV, *p* = 0.06). No significant differences were observed among TW, HFDW, and LFDW. A significant main effect of ROI was also found, *F* (3, 177) = 4.78, *p* = 0.003, *η_p_* ^2^ = 0.08. Pairwise comparisons indicated that P1 amplitude followed a roughly parietal‐dominant distribution, increasing from anterior to posterior regions and then decreasing. Specifically, the P1 amplitude in the parietal region (0.71 ± 0.28 µV) was significantly greater than that in the frontal (−0.19 ± 0.30 µV, *p* = 0.007), central (0.10 ± 0.27 µV, *p* = 0.02), and occipital regions (0.52 ± 0.30 µV, *p* = 0.03). In addition, the P1 amplitude in the central region was higher than in the frontal region (*p* = 0.004). Importantly, the interaction between word type and ROI was significant, *F* (9, 531) = 19.76, *p* < 0.001, *η_p_* ^2^ = 0.25. Simple effects analyses revealed that in the parietal region, the P1 amplitude for BW (2.03 ± 0.41 µV) was significantly higher than that for TW (0.28 ± 0.36 µV, *p* = 0.002), HFDW (0.17 ± 0.32 µV, *p* < 0.001), and LFDW (0.34 ± 0.35 µV, *p* < 0.001). No significant differences were found among target and distractor word types. Similarly, in the occipital region, the P1 amplitude for BW (2.00 ± 0.40 µV) was significantly greater than that for TW (0.05 ± 0.38 µV, *p* < 0.001), HFDW (−0.08 ± 0.35 µV, *p* < 0.001), and LFDW (0.12 ± 0.35 µV, *p* < 0.001). Again, no significant differences were observed among the target and distractor conditions. No significant differences in P1 amplitude were found among word types in the frontal and central regions.

**P1 Latency** A two‐way repeated‐measures ANOVA with factors word type (BW, TW, HFDW, LFDW) and ROI (frontal, central, parietal, occipital) was conducted on the peak latency of the P1 component (100–170 ms). No significant main effects were found for word type, *F* (3, 177) = 1.67, *p* = 0.18, *η_p_* ^2^ = 0.03, or ROI, *F* (3, 177) = 0.87, *p* = 0.46, *η_p_* ^2^ = 0.01. The interaction between word type and ROI was marginally significant, *F* (9, 531) = 1.72, *p* = 0.081, *η_p_* ^2^ = 0.03. Simple effects analysis showed that in the occipital region, the P1 latency for BW (136.97 ± 2.32 ms) was significantly longer than that for HFDW (128.10 ± 2.16 ms, *p* = 0.036), but did not differ significantly from TW (133.60 ± 2.09 ms) or LFDW (133.97 ± 2.32 ms). No significant differences in P1 latency were observed among word types in the frontal, central, or parietal regions (Figure 3).

**N1 Amplitude** A two‐way repeated‐measures ANOVA with factors word type (BW, TW, HFDW, LFDW) and ROI (frontal, central, parietal, occipital) was conducted on the mean amplitude of the N1 component (130–220 ms). The main effect of word type was not significant (Figure 3), *F* (3, 177) = 0.81, *p* = 0.49, *η_p_* ^2^ = 0.13. The main effect of ROI was marginally significant, *F* (3, 177) = 2.47, *p* = 0.06, *η_p_* ^2^ = 0.04. Pairwise comparisons indicated that the N1 amplitude in the occipital region (−0.11 ± 0.27 µV) was marginally more negative than in the central (0.51 ± 0.30 µV, *p* = 0.06) and parietal (0.09 ± 0.28 µV, *p* = 0.06) regions, but did not differ significantly from the frontal region. The interaction between word type and ROI was significant, *F* (9, 531) = 6.97, *p* < 0.001, *η_p_* ^2^ = 0.11. Simple effects analysis showed that in the occipital region, the N1 amplitude for TW (−0.40 ± 0.40 µV) was marginally more negative than for BW (0.61 ± 0.36 µV, *p* = 0.08), and the N1 amplitude for HFDW (−0.47 ± 0.30 µV) was significantly more negative than for BW (*p* = 0.03). No significant differences were observed among TW, HFDW, and LFDW. No significant differences among word types were found in the frontal, central, or parietal regions.

**N1 Latency**: A two‐way repeated‐measures ANOVA with factors word type (BW, TW, HFDW, LFDW) and ROI (frontal, central, parietal, occipital) was conducted on the peak latency of the N1 component (150–200 ms). A significant main effect of word type was found (Figure 3), *F* (3, 177) = 15.59, *p* < 0.001, *η_p_* ^2^ = 0.21. Pairwise comparisons revealed that the N1 latency for BW (177.33 ± 1.60 ms) was significantly longer than for TW (170.13 ± 1.75 ms, *p* < 0.001), HFDW (167.93 ± 1.42 ms, *p* < 0.001), and LFDW (169.37 ± 1.70 ms, *p* < 0.001), with no significant differences among the three divided‐attention conditions. The main effect of ROI was also significant, *F* (3, 177) = 13.47, *p* = 0.007, *η_p_* ^2^ = 0.19. Pairwise comparisons showed that N1 latency exhibited a posterior prolongation pattern, increasing from anterior to posterior regions. Specifically, latencies in the parietal (174.18 ± 1.42 ms) and occipital (174.16 ± 1.40 ms) regions were significantly longer than in the frontal (167.86 ± 1.75 ms, *p* = 0.001) and central regions (168.55 ± 1.66 ms, *p* = 0.001), with no significant difference between the parietal and occipital regions or between the frontal and central regions. The word type × ROI interaction was significant, *F* (9, 531) = 9.09, *p* < 0.001, *η_p_* ^2^ = 0.13. Simple effects analysis showed that in the parietal region, N1 latency for BW (183.47 ± 2.07 ms) was significantly longer than for TW (171.70 ± 1.88 ms, *p* < 0.001), HFDW (169.80 ± 1.50 ms, *p* < 0.001), and LFDW (171.77 ± 1.90 ms, *p* < 0.001), with no differences among the latter three conditions. Similarly, in the occipital region, BW (184.83 ± 1.95 ms) showed significantly longer latency than TW (171.47 ± 1.90 ms, *p* < 0.001), HFDW (169.30 ± 1.54 ms, *p* < 0.001), and LFDW (171.03 ± 1.87 ms, *p* < 0.001). No significant differences were observed among the three divided‐attention conditions. In the central region, BW (172.33 ± 2.22 ms) showed marginally longer latency than HFDW (166.73 ± 1.84 ms, *p* = 0.089), but did not differ significantly from TW (168.30 ± 2.24 ms) or LFDW (166.93 ± 1.91 ms). No significant differences were found among word types in the frontal region.

#### 3.2.2 ERP Components in Mid‐Encoding Stage

**P2 Amplitude**: A two‐way repeated‐measures ANOVA with factors word type (BW, TW, HFDW, LFDW) and ROI (frontal, central, parietal, occipital) was conducted on the mean amplitude of the P2 component (200–300 ms). The main effect of word type was not significant (Figure 3), *F* (3, 177) = 1.03, *p* = 0.38, *η_p_* ^2^ = 0.02, nor was the main effect of ROI, *F* (3, 177) = 1.68, *p* = 0.17, *η_p_* ^2^ = 0.03. However, the word type × ROI interaction was significant, *F* (9, 531) = 6.53, *p* < 0.001, *η_p_* ^2^ = 0.10. Simple effects analysis revealed that in the central region, the P2 amplitude for LFDW (1.90 ± 0.48 µV) was marginally higher than for HFDW (1.15 ± 0.42 µV, *p* = 0.05). No significant differences were observed between BW and TW, or among word types in the frontal, parietal, or occipital regions.

**P2 Latency**: A two‐way repeated‐measures ANOVA with factors word type and ROI was conducted on the peak latency of the P2 component (200–300 ms). The main effect of word type was significant (Figure 3), *F* (3, 177) = 3.57, *p* = 0.015, *η_p_* ^2^ = 0.06. Pairwise comparisons indicated that P2 latency for BW (267.88 ± 4.29 ms) was significantly longer than for LFDW (252.93 ± 4.58 ms, *p* = 0.051), but did not differ significantly from TW (262.83 ± 4.58 ms) or HFDW (257.83 ± 4.33 ms). No significant differences were found among TW, HFDW, and LFDW. The main effect of ROI was also significant, *F* (3, 177) = 42.98, *p* < 0.001, *η_p_* ^2^ = 0.42. Pairwise comparisons revealed a progressive increase in P2 latency from anterior to posterior regions: latencies in the occipital (274.39 ± 3.66 ms) and parietal (272.15 ± 3.97 ms) regions were significantly longer than those in the frontal (244.48 ± 3.80 ms, *p* < 0.001) and central (250.47 ± 4.08 ms, *p* < 0.001) regions. No significant differences were found between the occipital and parietal regions or between the frontal and central regions. The word type × ROI interaction was significant, *F* (9, 531) = 3.75, *p* < 0.001, *η_p_* ^2^ = 0.06. Simple effects analyses showed the following: Occipital region: P2 latency for TW (282.07 ± 5.45 ms) was significantly longer than for LFDW (262.87 ± 5.09 ms, *p* = 0.014). Latency for BW (278.57 ± 4.33 ms) was marginally longer than for LFDW (*p* = 0.090). No significant differences were found among BW, TW, and HFDW. Parietal region: P2 latency for TW (279.03 ± 5.78 ms) was significantly longer than for LFDW (259.73 ± 5.26 ms, *p* = 0.003). Latency for BW (275.80 ± 4.55 ms) and HFDW (274.03 ± 5.70 ms) was marginally longer than for LFDW (*p*s = 0.072 and 0.084, respectively). No significant differences were found among BW, TW, and HFDW. Central region: P2 latency for BW (260.27 ± 5.70 ms) was marginally longer than for HFDW (243.97 ± 5.46 ms, *p* = 0.085), but did not differ significantly from TW (250.10 ± 5.89 ms) or LFDW (247.53 ± 5.44 ms). No other differences were significant. Frontal region: P2 latency for BW (256.90 ± 5.79 ms) was significantly longer than for HFDW (239.27 ± 5.05 ms, *p* = 0.038) and marginally longer than for TW (240.13 ± 5.25 ms, *p* = 0.082). No significant differences were observed between BW and LFDW (241.60 ± 4.85 ms), or among TW, HFDW, and LFDW.

**N2 Amplitude** A two‐way repeated‐measures ANOVA with factors word type (BW, TW, HFDW, LFDW) and ROI (frontal, central, parietal, occipital) was conducted on the mean amplitude of the N2 component (250–350 ms). The main effect of word type was not significant (Figure 3), *F* (3, 177) = 1.81, *p* = 0.91, *η_p_* ^2^ = 0.003. The main effect of ROI was significant, *F* (3, 177) = 7.75, *p* < 0.001, *η*p^2^ = 0.12. Pairwise comparisons showed that N2 amplitude in the frontal region (0.25 ± 0.48 µV) was significantly more negative than in the parietal region (1.59 ± 0.37 µV, *p* = 0.006) and marginally more negative than in the occipital region (1.47 ± 0.36 µV, *p* = 0.06). N2 amplitude in the central region (0.57 ± 0.43 µV) was also significantly more negative than in the parietal region (*p* = 0.017). No significant differences were found between the frontal and central regions or between the parietal and occipital regions. The word type × ROI interaction was significant, *F* (9, 531) = 3.09, *p* = 0.001, *η_p_* ^2^ = 0.05. However, simple effects analyses revealed no significant differences in N2 amplitude among word types within any ROI.

**N2 Latency**: A two‐way repeated‐measures ANOVA with factors word type and ROI was conducted on the peak latency of the N2 component (250–350 ms). The main effect of word type was significant (Figure 3), *F* (3, 177) = 18.60, *p* < 0.001, *η_p_* ^2^ = 0.24. Pairwise comparisons indicated that the N2 latency for BW (367.44 ± 6.37 ms) was significantly longer than for TW (310.99 ± 5.34 ms, *p* < 0.001), LFDW (340.83 ± 6.96 ms, *p* = 0.008), and HFDW (337.48 ± 7.08 ms, *p* = 0.007). Similarly, N2 latency for TW was significantly shorter than for BW (*p* < 0.001), LFDW (*p* < 0.001), and HFDW (*p* = 0.005). No significant difference in latency was observed between LFDW and HFDW. The main effect of ROI was not significant, *F* (3, 177) = 0.11, *p* = 0.96, *η_p_* ^2^ = 0.002. However, the word type × ROI interaction was significant, *F* (9, 531) = 4.01, *p* < 0.001, *η_p_* ^2^ = 0.06. Simple effects analyses revealed the following: Frontal region: N2 latency for BW (367.63 ± 7.37 ms) was significantly longer than for TW (319.87 ± 6.25 ms, *p* < 0.001), LFDW (329.33 ± 7.63 ms, *p* < 0.001), and HFDW (336.70 ± 7.54 ms, *p* = 0.006). Central region: N2 latency for BW (369.17 ± 7.44 ms) was significantly longer than for TW (315.23 ± 6.12 ms, *p* < 0.001), LFDW (331.07 ± 7.40 ms, *p* < 0.001), and HFDW (338.67 ± 7.81 ms, *p* = 0.011). Parietal region: N2 latency for TW (302.30 ± 6.06 ms) was significantly shorter than for BW (367.70 ± 7.85 ms, *p* < 0.001), LFDW (349.33 ± 8.53 ms, *p* < 0.001), and HFDW (338.60 ± 8.91 ms, *p* = 0.002). Occipital region: N2 latency for TW (306.57 ± 6.61 ms) was significantly shorter than for BW (365.27 ± 8.03 ms, *p* < 0.001), LFDW (353.57 ± 8.38 ms, *p* < 0.001), and HFDW (335.93 ± 8.95 ms, *p* = 0.014).

#### 3.2.3 ERP Components in Late Encoding Stage

**P3 Amplitude** A two‐way repeated‐measures ANOVA with factors word type (BW, TW, HFDW, LFDW) and ROI (frontal, central, parietal, occipital) was conducted on the mean amplitude of the P3 component (300–650 ms). The main effect of word type was significant (Figure 3), *F*(3, 177) = 10.26, *p* < 0.001, *η_p_* ^2^ = 0.15. Pairwise comparisons showed that the P3 amplitude for TW (5.15 ± 0.66 µV) was significantly larger than for BW (2.82 ± 0.71 µV, *p* = 0.001), HFDW (2.56 ± 0.57 µV, *p* < 0.001), and LFDW (2.97 ± 0.50 µV, *p* < 0.001). No significant differences were found among BW, HFDW, and LFDW. The main effect of ROI was not significant, *F*(3, 177) = 0.69, *p* = 0.56, *η_p_* ^2^ = 0.01. However, the word type × ROI interaction was significant, *F*(9, 531) = 19.76, *p* < 0.001, *η_p_* ^2^ = 0.25. Simple effects analyses revealed the following: Central region: P3 amplitude for TW (4.65 ± 0.65 µV) was significantly larger than for HFDW (2.52 ± 0.60 µV, *p* = 0.003) and marginally larger than for LFDW (3.24 ± 0.57 µV, *p* = 0.086), but did not differ significantly from BW (3.32 ± 0.66 µV, *p* = 0.24). No significant differences were found among BW, HFDW, and LFDW. Parietal region: P3 amplitude for TW (6.23 ± 0.70 µV) was significantly larger than for BW (2.55 ± 0.74 µV, *p* < 0.001), HFDW (2.40 ± 0.50 µV, *p* < 0.001), and LFDW (2.75 ± 0.45 µV, *p* < 0.001). No significant differences were found among the three non‐target conditions. Occipital region: P3 amplitude for TW (5.81 ± 0.72 µV) was significantly larger than for BW (2.10 ± 0.81 µV, *p* < 0.001), HFDW (2.18 ± 0.50 µV, *p* < 0.001), and LFDW (2.29 ± 0.46 µV, *p* < 0.001). No significant differences were observed among BW, HFDW, and LFDW. Frontal region: No significant differences in P3 amplitude were observed among the four word types.

**P3 Latency** A two‐way repeated‐measures ANOVA with factors word type and ROI was conducted on the peak latency of the P3 component (300–650 ms). The main effect of word type was significant (Figure 3), *F*(3, 177) = 5.38, *p* = 0.001, *η_p_* ^2^ = 0.084. Pairwise comparisons showed that the P3 latency for BW (526.57 ± 12.12 ms) and TW (513.53 ± 8.82 ms) was significantly longer than for HFDW (477.02 ± 11.41 ms, *p* = 0.010–0.012), but did not differ significantly from LFDW (500.48 ± 10.42 ms). No significant differences were found between BW and TW, nor between LFDW and HFDW. The main effect of ROI was significant, *F*(3, 177) = 17.46, *p* < 0.001, *η_p_* ^2^ = 0.23. Pairwise comparisons showed that P3 latency decreased progressively from anterior to posterior sites: latencies in the frontal (526.67 ± 9.11 ms) and central regions (522.83 ± 8.89 ms) were significantly longer than in the parietal (485.43 ± 8.45 ms) and occipital regions (482.66 ± 8.73 ms, *p* < 0.001). No significant differences were found between frontal and central regions or between parietal and occipital regions. The word type × ROI interaction was not significant, *F*(9, 531) = 0.98, *p* = 0.45, *η_p_* ^2^ = 0.016.

### 3.3 EEG Results in Recognition Stage

**FIGURE 4:**
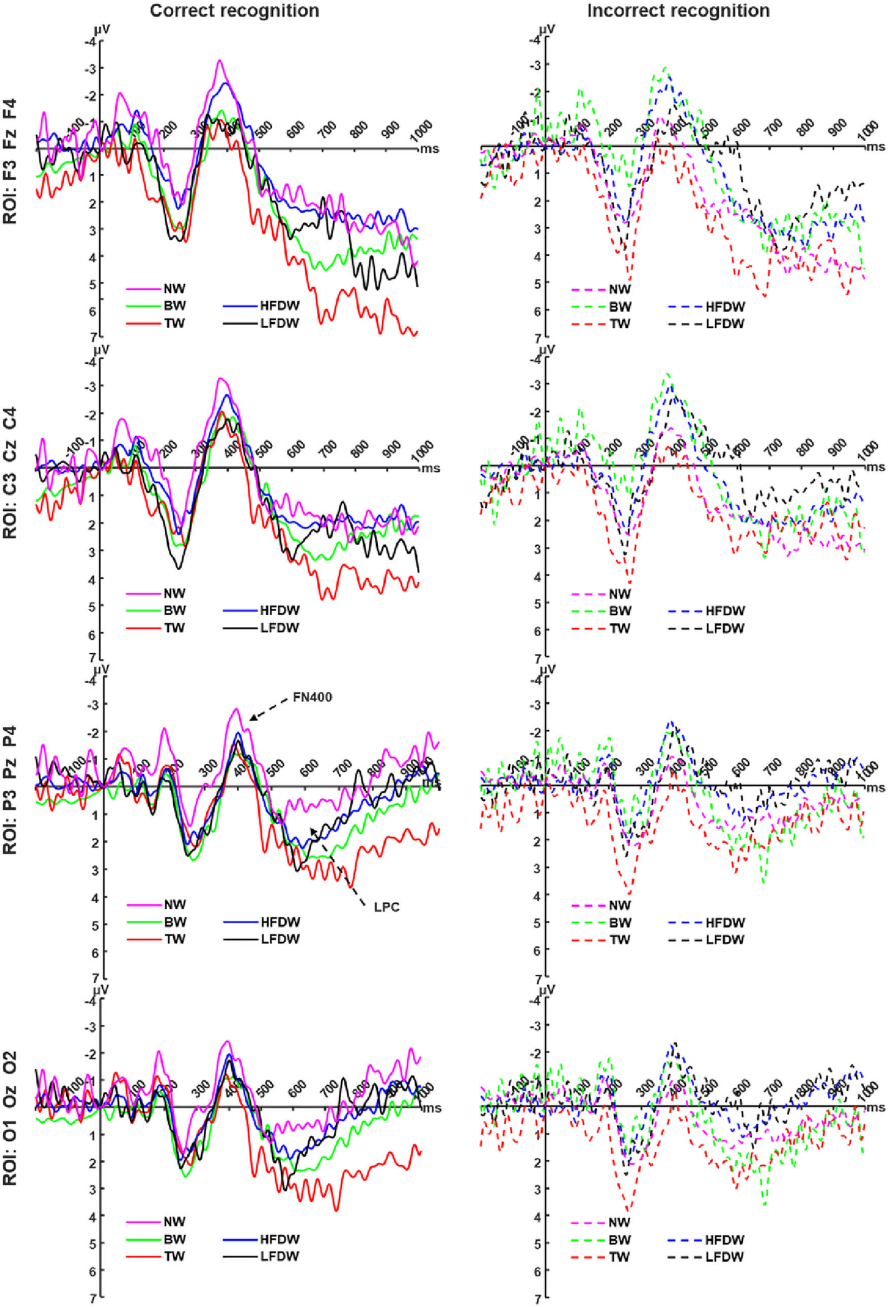
Grand‐averaged ERP waveforms during the recognition stage. The left panel shows the grand‐averaged ERP waveforms for the five word types (BW, TW, HFDW, LFDW, and NW) under the correct recognition condition across four regions of interest (ROI1–ROI4), while the right panel shows the corresponding waveforms under the incorrect recognition condition. The *x*‐axis represents time (ms), and the y‐axis represents ERP amplitude (µV). BW, baseline words; HFDW, high‐frequency distractor words; LFDW, low‐frequency distractor words; NW, new words; TW, target words.

**FN400 Old‐New Effect**: A three‐way repeated‐measures ANOVA with factors recognition outcome (correct, incorrect) × word type (BW, TW, HFDW, LFDW, NW) × ROI (frontal, central, parietal, occipital) was conducted on the mean amplitude of the FN400 (300–500 ms). The main effect of ROI was marginally significant (Figure 4), *F*(3, 177) = 2.28, *p* = 0.081, *η_p_* ^2^ = 0.04. Pairwise comparisons indicated that FN400 amplitude in the central region (−0.92 ± 0.42 µV) was significantly more negative than in the frontal (−0.50 ± 0.46 µV, *p* = 0.031), parietal (−0.22 ± 0.47 µV, *p* = 0.027), and occipital regions (−0.16 ± 0.46 µV, *p* = 0.038), with no significant differences among the latter three regions. The three‐way interaction among recognition outcome, word type, and ROI was significant, *F*(12, 708) = 0.62, *p* = 0.83, *η_p_* ^2^ = 0.01. Simple‐effects analyses revealed the following patterns: Under correct recognition, in the frontal region, FN400 amplitudes elicited by NW (−1.47 ± 0.87 µV) and HFDW (−1.24 ± 0.49 µV) were marginally more negative than those elicited by TW (0.33 ± 0.86 µV, *p* = 0.055–0.057). In the parietal region, NW (−1.17 ± 0.72 µV) elicited marginally more negative FN400 amplitudes than TW (0.16 ± 0.76 µV, *p* = 0.091). Under incorrect recognition, in the frontal region, FN400 amplitudes elicited by BW (−1.54 ± 1.06 µV) were marginally more negative than those for TW (0.98 ± 0.84 µV, *p* = 0.072). HFDW (−1.22 ± 0.55 µV) elicited significantly more negative amplitudes than TW (0.98 ± 0.84 µV, *p* = 0.018) and NW (0.21 ± 0.68 µV, *p* = 0.022). In the central region, BW (−1.91 ± 0.94 µV) were marginally more negative than TW (0.25 ± 0.73 µV, *p* = 0.078), and HFDW (−1.46 ± 0.58 µV) were significantly more negative than both TW (*p* = 0.053) and NW (−0.25 ± 0.58 µV, *p* = 0.020). In the parietal region, HFDW (−0.90 ± 0.53 µV) elicited significantly more negative amplitudes than TW (0.99 ± 0.78 µV, *p* = 0.028) and NW (0.20 ± 0.52 µV, *p* = 0.020). In the occipital region, HFDW (−0.85 ± 0.52 µV) were significantly more negative than both TW (1.07 ± 0.82 µV, *p* = 0.025) and NW (0.25 ± 0.51 µV, *p* = 0.028).

**LPC Old‐New Effect**: A three‐way repeated‐measures ANOVA with factors recognition outcome (correct, incorrect) × word type (BW, TW, HFDW, LFDW, NW) × ROI (frontal, central, parietal, occipital) was conducted on the mean amplitude of the LPC (500–1000 ms). The main effect of ROI was significant (Figure 4), *F*(3, 177) = 12.54, *p* < 0.001, *η_p_* ^2^ = 0.18. Pairwise comparisons revealed that LPC amplitude decreased from anterior to posterior sites: the frontal region (3.50 ± 0.99 µV) showed significantly larger amplitudes than the parietal (0.85 ± 0.51 µV, *p* = 0.004) and occipital regions (0.67 ± 0.49 µV, *p* = 0.003); the central region (2.27 ± 0.57 µV) also showed significantly larger amplitudes than both parietal and occipital regions (*p* < 0.001). No significant differences were found between frontal and central, or between parietal and occipital regions. The main effect of word type was marginally significant, *F*(4, 236) = 2.34, *p* = 0.056, *η_p_^2^* = 0.04, though no pairwise differences reached significance. The three‐way interaction among recognition outcome, word type, and ROI was not significant, *F*(12, 708) = 0.71, *p* = 0.75, *η_p_* ^2^ = 0.02. Simple‐effects analysis indicated that under correct recognition, in the frontal region, LPC amplitudes for TW (5.80 ± 1.23 µV) were significantly greater than for HFDW (2.55 ± 0.84 µV, *p* = 0.046) and marginally greater than for NW (2.65 ± 1.11 µV, *p* = 0.084). In the parietal region, LPC amplitudes for TW (2.42 ± 0.88 µV) were marginally greater than for NW (−0.33 ± 0.84 µV, *p* = 0.073). Under incorrect recognition, no significant LPC differences were found across word types in any ROI.

## 4 Discussion

### 4.1 Behavioral Results

The one‐way repeated‐measures ANOVA on encoding‐stage detection accuracy showed significantly lower accuracy for TW compared to the other three word types. Despite this difference, detection accuracy in the TW condition remained high (>95%), likely due to the active response requirement for TWs versus the passive or inhibitory demands of other conditions, confirming consistent participant engagement. During the recognition stage, analyses of ORA, CRA, and discriminability index (*d′*) across the four word types (BW, TW, HFDW, LFDW) revealed that recognition accuracy for TW was significantly higher than that for HFDW, LFDW, and BW. This result is consistent with the behavioral hypothesis and provides clear evidence of an ABE in the current study. Moreover, BW were recognized with significantly higher accuracy than both HFDW and LFDW. This memory advantage for BW over distractors was consistently observed across measures of ORA, CRA, and discriminability index (*d′*). Superficially, this pattern aligns with the distractor inhibition hypothesis, which posits that the active suppression of a distractor stimulus can impair memory encoding for concurrently presented information. As mentioned in the introduction, previous research has consistently demonstrated that selective attention operates through a dual mechanism involving both the activation of target information and the inhibition of distracting information (Meng et al. 2019; Sisk and Lee 2024). In line with this framework, the current behavioral results suggest that the ABE arises from the combined action of target enhancement and distractor inhibition. Contrary to our hypothesis, no significant differences in ORA, CRA, or the discriminability index (*d′*) were observed between HFDW and LFDW, suggesting that the occurrence frequency of distractor words did not influence their memory performance.

### 4.2 ERP Results in Early Encoding Stage

Statistical analyses revealed that differences in the early ERP components P1 and N1 emerged primarily in the parietal and occipital regions and were mainly observed between BW and the other word types. Specifically, compared with TW, LFDW, and HFDW, BW elicited larger P1 amplitudes, smaller N1 amplitudes, and longer peak latencies for both components. No significant differences in the mean amplitude or latency of P1 and N1 were observed among TW, HFDW, and LFDW. The absence of amplitude or latency differences between TW, HFDW, and LFDW is consistent with previous findings (Lin et al. 2020). One possible explanation is that, under the dual‐task paradigm used in the present study, participants engaged in comparable visual‐perceptual recognition processes for both target and distractor stimuli. Both required perceptual identification of the probe stimulus before a corresponding judgment and response could be made. Consequently, target and distractor words did not differ in early perceptual processing components (Lin et al. 2020). In contrast to Lin et al. (2020), however, the present study found that BW, relative to TW, HFDW, and LFDW, elicited more positive P1 amplitudes, smaller N1 amplitudes, and longer latencies for both components. Previous research has shown that P1 and N1 are closely associated with early, automatic selective attention to the perceptual features of stimuli and are modulated by their physical attributes—such as color and shape (Yun et al. 2011)—as well as by task difficulty (Luck et al. 1989). Compared with complex tasks (e.g., invalid cues in the Posner cueing paradigm), simpler tasks (e.g., valid cues) tend to elicit larger P1 amplitudes, reflecting a higher degree of automaticity (Hillyard and Anllo‐Vento 1998; Luck et al. 1989). Therefore, the more positive P1 amplitude and longer latency observed for BW in the present study may be attributable to the fact that, in the baseline condition, background information was presented alone and not paired with any probe stimulus. As a result, the baseline condition differed from both target and distractor conditions in physical characteristics and task complexity, exhibiting greater specificity in early perceptual processing.

### 4.3 ERP Results in Mid‐Encoding Stage

Statistical analyses revealed that the differences in the mid‐encoding stage ERP components (P2 and N2) were manifested primarily in component latencies rather than amplitudes. Specifically, for the P2 component, latency patterns differed across brain regions: in frontal and central areas, TW showed the shortest latency, while BW exhibited the longest; in parietal and occipital regions, BW continued to display the longest latency. For the N2 component, latency differences were consistent across all four regions of interest, with TW having the shortest latency and BW the longest. Previous studies suggest that the P2 is associated with attention maintenance and stimulus categorization (Hillyard and Anllo‐Vento 1998; Martin and Potts 2004), whereas the N2 is implicated in conflict monitoring and response inhibition (Donkers and van Boxtel 2004; Larson et al. 2014). Given the distinct characteristics of the experimental conditions—TW required a keypress response to the target digit “7”, distractor words (HFDW and LFDW) were presented with task‐irrelevant digits requiring response inhibition, and BW were presented without any digit and thus involved only the memorization task—the observed latency patterns suggest that P2 latency likely reflects the discrimination of target stimuli, whereas N2 latency may index the inhibition of non‐target distractors, consistent with the distractor inhibition hypothesis. This interpretation is further corroborated by the superior recognition performance for TW, as well as the better recognition accuracy for BW compared to HFDW and LFDW in the subsequent recognition stage. Collectively, these findings suggest that during the mid‐encoding stage, the brain simultaneously engages in rapid discrimination of target stimuli and active inhibition of non‐target distractors, thereby facilitating the allocation of attentional resources in subsequent late encoding stage. Moreover, the lack of significant differences in ERP amplitude, latency, or behavioral memory performance between HFDW and LFDW implies that distractor inhibition was not influenced by the frequency of distractor words.

### 4.4 ERP Results in Late Encoding Stage

Statistical analyses revealed that TW elicited significantly larger late P3 amplitudes than BW, HFDW, and LFDW, with no significant differences among the latter three conditions. This pattern is consistent with previous ERP studies on the ABE (Jing 2015; Lin 2019a; Lin et al. 2020), which have consistently reported larger late P3 amplitudes for target stimuli relative to distractor stimuli. However, interpretations of the functional significance of the P3 component have varied: some researchers have proposed that P3 reflects cognitive resource allocation (Jing 2015), whereas others have argued that it reflects memory updating processes (Lin 2019a). In the present study, the occurrence probabilities of TW, BW, and LFDW were identical; however, only target words elicited significantly larger P3 amplitudes. This finding does not support Lin's interpretation that P3 reflects the novelty of probe stimuli. Instead, it aligns more closely with Jing's view that P3 reflects target enhancement triggered by the detection of target stimuli. Again, contrary to our hypothesis that LFDW would elicit larger P3 amplitudes than HFDW, no significant differences in P3 amplitude were observed in the present study. This finding suggests that the occurrence probability of distractor stimuli does not modulate the allocation of attentional resources.

It should be noted that in Lin's study, the occurrence probability of distractor stimuli in the high‐frequency target condition was much lower than that of target stimuli. Previous research has demonstrated that, due to the principle of cognitive economy, individuals tend to treat low‐probability stimuli as potential targets (Siqi Zheng and Huang 2022). Thus, the larger P3 amplitude observed for low‐frequency distractor stimuli in Lin's high‐frequency target group may have resulted from participants mistakenly treating these distractors as targets, effectively reversing task instructions (Siqi Zheng and Huang 2022). From this perspective, the P3 effect in Lin's experiment still reflects target enhancement driven by target detection processing rather than novelty per se.

Additionally, statistical analyses showed that TW and BW exhibited longer P3 latencies than distractor words, and that anterior brain regions (frontal and central) showed longer latencies than posterior regions (parietal and occipital). The prolonged P3 latency for BW may be a continuation of the delayed processing observed in earlier visual recognition stages. The longer P3 latency and duration for TW may reflect the sustained impact of generalized attentional enhancement induced by target detection during the encoding stage. This enhancement likely facilitated greater allocation of attentional resources to background words (TW) that co‐occurred with target stimuli, promoting elaborate processing, such as rehearsal and storage, during the late encoding stage (Lin et al. 2020).

### 4.5 ERP Old–New Effect During the Recognition Stage

ERP studies on recognition memory have consistently shown that repeated stimuli elicit more positive‐going waveforms than novel stimuli—a phenomenon referred to as the ERP old–new effect (Lucas et al. 2012). This effect is primarily associated with two components: the frontal negative FN400 and the LPC. The FN400 is thought to reflect item familiarity (Meng and Guo 2009) and perceptual fluency (Guo et al. 2013), whereas the LPC is typically associated with recollection or the retrieval of item‐specific contextual details (Meng and Guo 2009; Rugg and Curran 2007). In the present study, under conditions of correct recognition, ERPs elicited by TW were more positive‐going than those elicited by NW. Specifically, NW evoked a more negative FN400 than TW, while TW induced a more positive LPC than NW—both characteristic patterns of the old–new effect. Given that ERP differences between TW and other word types during the encoding stage primarily emerged and persisted into the late stage, we infer that the recognition advantage for TW originates from generalized attentional enhancement triggered by target detection during the encoding stage. This attentional enhancement likely increased resource allocation and promoted elaborate processing of background words, resulting in heightened perceptual fluency and improved memory performance at recognition stage. Furthermore, this enhancement may have facilitated rehearsal and storage of these words during the late encoding stage. Consequently, these encoding‐related advantages were reflected in the retrieval‐related ERP components—specifically, reduced FN400 and enhanced LPC effects—during the recognition stage. In addition, we did not observe significant differences in the FN400 or LPC old/new effects between HFDW and LFDW. This indicates that, although we manipulated distractor frequency to probe its potential role, this variable did not systematically modulate the neural correlates of familiarity‐based or recollection‐based retrieval success for distractor‐paired items.

## 5 Conclusions

**FIGURE 5:**
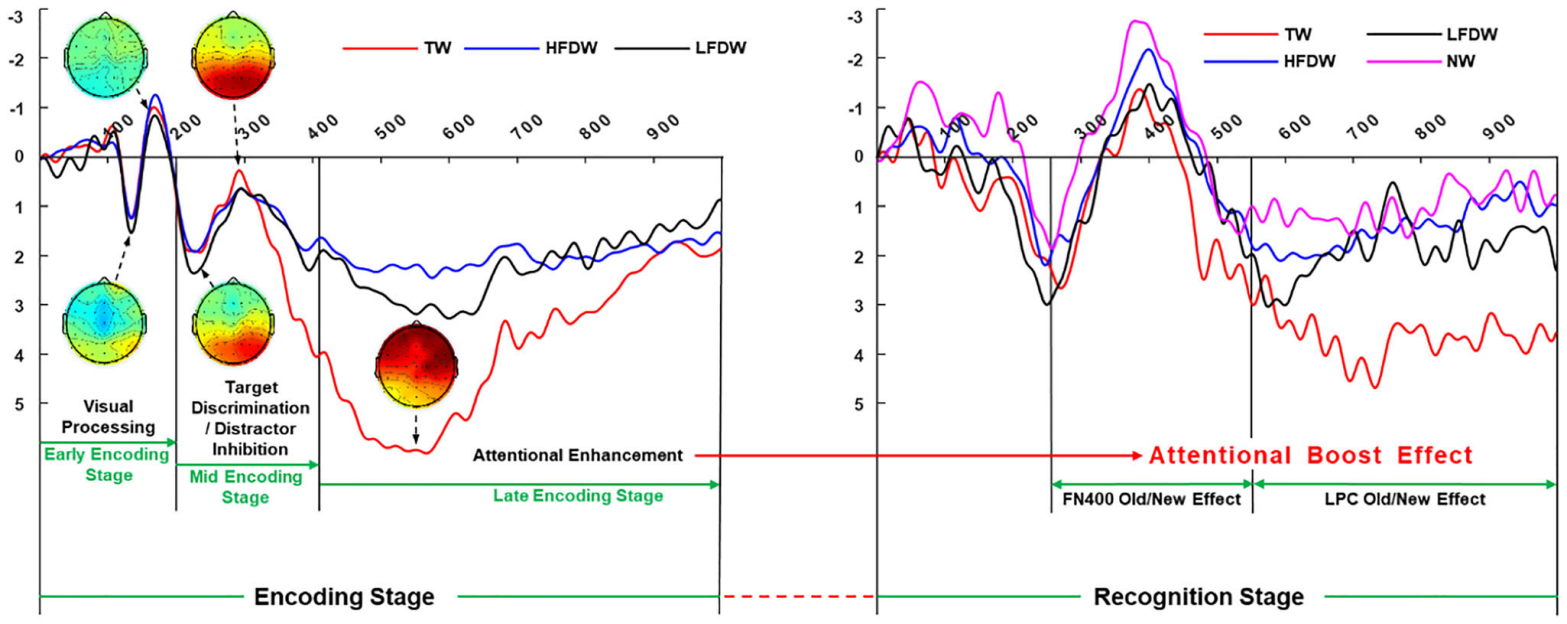
Neural dynamics across encoding and recognition stages of the attentional boost effect. HFDW, high‐frequency distractor words; LFDW, low‐frequency distractor words; NW, new words; TW, target words.

Based on the ERP findings across the encoding and recognition stages, a tentative model of the neural dynamics of the ABE can be proposed (Figure 5). Early Encoding Stage: The brain's primary task is to perform visual recognition of incoming stimuli. At this stage, target and distractor stimuli undergo similar perceptual identification and judgment processes, resulting in comparable early perceptual components (P1 and N1). In contrast, BW—presented alone without probe stimuli—elicited larger P1 and smaller N1 amplitudes, reflecting their higher perceptual specificity. Mid‐Encoding Stage: Building on early perceptual processing, the brain simultaneously engages in rapid discrimination of target stimuli (P2) and active inhibition of non‐target distractors (N2), which lays the groundwork for the subsequent allocation of attentional resources. Late Encoding Stage: a significantly greater allocation of attentional resources to target stimuli promotes more elaborate memory processing, such as active rehearsal and consolidation. Such enhanced encoding establishes a more robust memory trace, thereby leading to superior recognition performance for target stimuli in the subsequent memory test. Recognition stage: The attentional enhancement (or elaborative processing) afforded to target stimuli during the encoding stage appears to persist into the recognition stage. Behaviorally, this sustained benefit was manifested as significantly better memory performance (higher ORA, CRA, and discriminability index *d′*) for target stimuli. Neutrally, it was reflected in the classic ERP old/new effect: NW evoked a more negative‐going FN400 than TW, whereas TW elicited a more positive‐going LPC than NW.

As discussed above, selective attention operates through two complementary mechanisms: selective focus on target information and selective inhibition of distractor stimuli. Accordingly, the ABE may arise either from enhanced processing of stimuli presented concurrently with targets (the target enhancement hypothesis) or from impaired memory for background information due to distractor suppression (the distractor inhibition hypothesis). Combined with the neural dynamics model of the ABE proposed above and the behavioral results that recognition accuracy for TW was significantly higher than that for HFDW, LFDW, and BW, while BW were recognized with significantly higher accuracy than both HFDW and LFDW, we conclude that, in the present study, the ABE arises from the combined action of target enhancement and distractor inhibition.

## 6 Limitations and Future Directions

Although the present study provides valuable insights into the neural dynamics underlying the ABE, several limitations should be acknowledged. First, during the recognition stage, an unequal number of old words from each encoding condition was presented (HFDW: 48; BW, TW, LFDW: 12 each) to match their original presentation frequency in encoding. While this may introduce a potential confound due to trial number imbalance, our core analyses focus on between‐condition comparisons of memory and ERP effects, not absolute response rates. All conditions contained sufficient trials for stable ERP averaging, and key effects emerged even in conditions with fewer trials, suggesting the imbalance did not drive the primary results. We acknowledge this as a methodological trade‐off, and future studies could employ a fully balanced design to further validate our conclusions. Secondly, the current ERP design provides excellent temporal resolution but limited spatial precision. Future studies combining EEG with functional neuroimaging (e.g., fMRI or fNIRS) could clarify the neural networks involved in attentional enhancement, particularly the interactions between frontoparietal attention systems and medial temporal memory regions. Moreover, this study primarily focused on behavioral and electrophysiological correlates of target enhancement. Future research should further investigate individual differences—such as cognitive capacity, attentional control, and motivational factors—that might modulate the magnitude of the ABE.

Beyond its theoretical contribution to understanding attention–memory interactions, the current findings have potential clinical implications. The demonstration that target detection can transiently enhance memory encoding suggests a possible avenue for cognitive training and rehabilitation. For instance, attentional facilitation mechanisms could be leveraged to improve memory and attentional control in individuals with neurological or psychiatric conditions characterized by attentional deficits, such as attention deficit hyperactivity disorder (ADHD), mild cognitive impairment (MCI), or traumatic brain injury (TBI). Future clinical studies may explore whether controlled modulation of attentional engagement can strengthen memory encoding and cognitive efficiency in these populations.

## Author Contributions

**Xintong Chen**: conceptualization, data curation, formal analysis, methodology, writing – review and editing. **Xuan Lyu**: formal analysis, methodology, visualization, writing – original draft. **Li Zhu**: data curation. **Qin Cui**: data curation. **Qing Yang**: data curation. **Xinglin Li**: visualization, writing – review and editing. **Kaiye Xiang**: visualization, writing – review and editing. **Chun Zheng**: supervision, validation, visualization, writing – review and editing. **Chao Fu**: conceptualization, formal analysis, funding acquisition, methodology, project administration, writing –original draft, writing – review and editing.

## Funding

This work was supported by the National Natural Science Foundation of China (Grant No. 32260203), the General Program of the Qinghai Province Science and Technology Plan (Grant No. 2025‐ZJ‐957 M), and the Major Science and Technology Special Projects of Qinghai Province (Grant No. 2024‐SF‐A2).

## Ethics Statement

This study was approved by the Ethics Committee of Qinghai Normal University (Approval No. QHNU2024LS‐12, April 7, 2024).

## Consent

Written informed consent was obtained from all participants prior to their inclusion in the study.

## Conflicts of Interest

The authors declare no conflicts of interest.

## Data Availability

The data supporting the findings of this study are available from the corresponding author upon reasonable request.
